# Singlet Oxygen Produced by Aspalathin and Ascorbic Acid Leads to Fragmentation of Dihydrochalcones and Adduct Formation

**DOI:** 10.1021/acs.jafc.4c07633

**Published:** 2024-09-26

**Authors:** Vanessa K. Fokuhl, Emma L. Gerlach, Marcus A. Glomb

**Affiliations:** Institute of Chemistry, Food Chemistry, Martin-Luther-University Halle-Wittenberg, Kurt-Mothes-Str. 2, 06120 Halle/Saale, Germany

**Keywords:** dihydrochalcone, chalcone, oxidative fragmentation, singlet oxygen, aspalathin, phloretin, ascorbic acid, apple

## Abstract

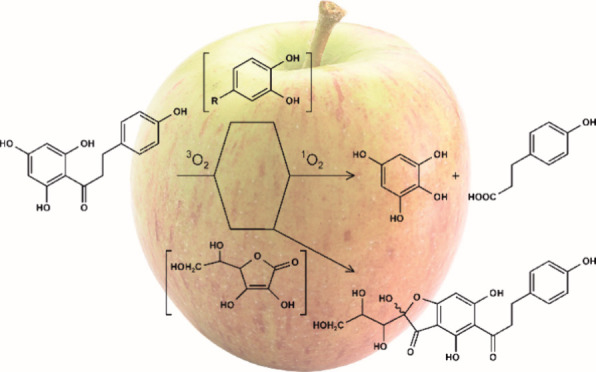

Singlet oxygen-mediated fragmentation of various dihydrochalcones and chalcones was reported. (Dihydro)cinnamic acids formed in the fragmentation showed a B-ring substitution pattern of the precursor (dihydro)chalcone. For the first time, the intrinsic generation of singlet oxygen by aspalathin and ascorbic acid under mild aqueous conditions (37 °C, pH 7.0) and exclusion of light was verified using HPLC-(+)-APCI-MS^2^ experiments. If a 4 molar excess of aspalathin or ascorbic acid was used, fragmentation of dihydrochalcones with monohydroxy and *o*-hydroxymethoxy B-ring substitution was induced up to 2 mol %, respectively. Incubations of the dihydrochalcone phloretin with ascorbic acid not only led to *p*-dihydrocoumaric acid but also to a novel ascorbyl adduct, which was isolated and identified as 2,4,6-trihydroxy-5-[3-(4-hydroxyphenyl)propanoyl]-2-[(1*R*, 2*S*)-1,2,3-trihydroxypropyl]-1-benzofuran-3(2*H*)-one. The impact of different structural elements on adduct formation was evaluated and verified to be a phloroglucinol structure linked to an acyl moiety. Formation of the ascorbyl adduct was shown to occur in apple puree when both ascorbic acid and phloretin were present at the same time.

## Introduction

It is well known that the consumption of a diet rich in fruits and vegetables has a benefit on human health.^1^ This value is associated with but not limited to the intake of dietary flavonoids, which are generated by plants as secondary metabolites for protection against abiotic stresses, such as UV-radiation, herbivores, pathogens, or climatic changes such as heat or drought.^2^ Due to their favorable impact on human health based on antioxidative, antimutagenic, anticarcinogenic, and anti-inflammatory properties flavonoids have gained increasing interest during the last decades.^3−6^ Quantitative relevant flavonoid classes in fruits and vegetables are flavonols, flavones, isoflavones, flavanones, flavan-3-ols and chalcones.^1^ The latter are found as dihydrochalcone glycosides in apple varieties^7^ which are one of the most popular fruits in Europe^8−10^ as fresh fruits and processed as juice or puree. However, during manufacturing apple dihydrochalcones are oxidized via enzymes such as polyphenol oxidases or by nonenzymatic pathways.^11,12^ In our previous studies we reported oxidative rearrangement-fragmentation reactions of the dihydrochalcones aspalathin and phloretin initiated by singlet oxygen.^12^ Both aspalathin and phloretin were fragmented when molybdate and hydrogen peroxide as exogenous singlet oxygen sources were added to the incubations. Aspalathin underwent a fragmentation reaction to the corresponding dihydrocaffeic acid even without the addition of molybdate and hydrogen peroxide. It was therefore concluded that aspalathin is able to intrinsically generate singlet oxygen under mild conditions and the absence of light. However, singlet oxygen itself was not directly measured. Commonly, singlet oxygen is verified indirectly by measuring specific oxidized products as in our previous study,^12^ directly by chemiluminescence at 1275 nm^13^ or by using chemical traps.^14^ The latter react with singlet oxygen to give specific endoperoxides that can be measured in trace amounts. In the literature, the formation of singlet oxygen by flavonoids has been demonstrated exclusively for photochemical conditions. To our knowledge, evidence for the intrinsic formation of singlet oxygen by plant phenols has not been reported for model reactions in the absence of light.

Besides their ability to generate reactive oxygen species, flavonoids have been shown to function as trapping agents of reactive dicarbonyl species^15^ which might have detrimental effects on aging and pathologic processes.^16^ Apples contain significant amounts of carbohydrates, such as sugars and ascorbic acid which can serve as precursor molecules for the generation of dicarbonyl compounds like dehydroascorbic acid, glyoxal, or methylglyoxal.^16,17^ In the past decades evidence has been given for the trapping mechanism by nucleophilic attack of the A-ring of flavonoids like catechin, quercetin, or phloretin and its glycoside phloridzin^15,18,19^ which can all be found in apple cultivars.^7^

Thus, the aim of this study was to reinvestigate the stability of dihydrochalcones in the presence of singlet oxygen with respect to the formation of specific oxidative fragmentation products. Most importantly, the generation of endogenously generated singlet oxygen by dihydrochalcones and by ascorbic acid was verified as the triggering reactive species. Furthermore, a novel phloretin ascorbyl adduct was isolated, including studies on the formation mechanism and the detection in apple puree.

## Materials and Methods

### Chemicals

All chemicals of the highest quality available were obtained from Sigma-Aldrich (Munich/Steinheim, Germany), Roth (Karlsruhe, Germany), ACROS Organics (Geel, Belgium), Merck (Darmstadt, Germany), Fluka (Taufkirchen, Germany), and VWR Chemicals (Darmstadt, Germany) unless otherwise indicated. For all experiments, ultrapure water (Ultra Clear, Siemens, Munich, Germany) was used. Apples (Nicoter) and commercial apple puree were purchased at local food stores.

### Aerated Phenol Incubations

0.5 mM phenol (aspalathin, phloretin, phloridzin, naringin-dihydrochalcone, neohesperidin-dihydrochalcone, naringenin, hesperetin, eriodictyol, xanthohumol, phloroglucinol, 2,4-dihydroxyacetophenone, 2,4-dihydroxybenzoic acid, 2,4,6-trihydroxyacetophenone, 2,4,6-trihydroxybenzoic acid, and 2,4,6-trihydroxytoluene) was dissolved in 2 mL of phosphate buffer (0.1 M, pH7) and incubated in screw cap vials. The only exception was xanthohumol, which was incubated in a 1:1 mixture of phosphate buffer (0.1 M, pH7) and methanol due to poor solubility. To some incubations, 2 mM aspalathin, 2 mM ascorbic acid, 2 mM dimethylnaphthalene-endoperoxide (DMN-EP), or 0.5 mM hydrogen peroxide was added as a singlet oxygen-generating substance. Incubations were kept in a shaker at 37 °C under the exclusion of light. The formation of cinnamic acids as fragmentation products was analyzed by gas chromatography with flame ionization detection (GC-FID) and coupled gas chromatography–mass spectrometry (GC-MS) after silylation. Phenols were analyzed using high-performance liquid chromatography with diode array detection (HPLC-DAD) and coupled high-performance liquid chromatography–mass spectrometry (HPLC-MS). Sample preparation was as follows: For HPLC analysis, an aliquot of the incubations was directly injected. For GC analysis, an aliquot of the sample was acidified with HCl, extracted with diethyl ether, and the solvent was removed under an argon atmosphere. The dried extracts were dissolved in 50 μL of pyridine, and 50 μL of *N,O*-bis(trimethylsilyl)acetamide with 5% trimethylchlorosilane was added. Samples were kept at room temperature for 1 h prior to injection into the GC system.

### Deaerated Polyphenol Incubations

The incubations were modified by adding 1 mM diethylenetriaminepentaacetic acid to the phosphate buffer. All solvents were degassed first in an ultrasonic bath for 15 min and then with helium for 20 min. Samples were incubated in screw-capped vials without air. Sample preparation was identical to aerated incubations where chelator and degassing were omitted.

### Synthesis of 1,4-Dimethylnaphthalene-1,4-endoperoxide (DMN-EP) and 9,10-Diphenylanthracene-9,10-endoperoxide (DPA-EP)

DMN-EP was synthesized as previously reported by Heymann.^20^ Before use, the composition of the reaction products was characterized by ^1^H-NMR. NMR data matched those reported by Wasserman.^21^ DMN-EP synthesis gave a mixture of about (1:1) consisting of DMN-EP and DMN. DPA-EP was synthesized in the same way as DMN-EP and was shown to be quantitatively converted to the corresponding DPA-EP. 1H-NMR spectra were comparable to data reported by Martinez-Agramunt^22^: (400 MHz, CDCl_3_) δ: 7.18 ppm (dt, ^3^*J* = 5.7 Hz, ^4^*J* = 3.6 Hz, 4H), 7.21 (dt, ^3^*J* = 5.7 Hz, ^4^*J* = 3.5 Hz, 4H), 7.54 (tt, ^3^*J* = 7.4 Hz, ^4^*J* = 1.4 Hz, 2H), 7.63 (m, ^3^*J* = 7.4 Hz, ^3^*J* = 7.1 Hz, ^4^*J* = 1.3 Hz, 4H), 7.71 (dt, ^3^*J* = 7.1 Hz, ^4^*J* = 1.3 Hz, 4H).

### Detection of Singlet Oxygen

In order to verify the generation of singlet oxygen in the reaction of aspalathin or ascorbic acid (20 mM) with 0.5 mM phloretin or other dihydrochalcones, we chose 0.1 mM DPA as a trapping reagent. Samples were incubated in a 1:1 mixture of phosphate buffer (0.1 M, pH7) and acetonitrile due to the poor solubility of DPA. Incubations were allowed to react for 24 h at 37 °C under aeration. Blanks without aspalathin or ascorbic acid were conducted in the same way. Detection was carried out using coupled analytical HPLC-MS. Samples were directly injected.

### Isolation of Phloretin Ascorbyl Adduct and 2,4,6-Trihydroxyacetophenone Ascorbyl Adduct

For isolation of the phloretin ascorbyl adduct, the incubation was upscaled to 2 L (0.5 mM phloretin, 2 mM ascorbic acid, phosphate buffer 0.1 M/pH 7) and extracted twice with 800 mL of diethyl ether to remove unreacted phloretin. After acidification with 6 M HCl to pH 1 the adduct was extracted twice with 800 mL of ethyl acetate. Ethyl acetate was evaporated under reduced pressure at 30 °C and the solid was dissolved in 2 mL of methanol/water (4:6, v/v) with 0.8 μL mL^–1^ formic acid for purification via preparative reversed-phase chromatography. For isolation of the 2,4,6-trihydroxyacetophenone ascorbyl adduct, incubations were done with 2,4,6-trihydroxyacetophenone, the adduct preseparated by flash chromatography (RP18, 40–63 μm, methanol/water (1/1, v/v), fractions containing the adduct combined and evaporated, and the final residue taken up in 2 mL of methanol/water (1/9, v/v) with 0.8 μL mL^–1^ formic acid for final preparative reversed-phase chromatography. Chromatographic fractions were monitored by HPLC-DAD.

### Preparative Reversed-phase Chromatography

The glass column (Merck, LOBAR LiChroprep RP-18 (31.0 cm × 2.5 cm, 40–63 μm), Darmstadt, Germany) was connected to a Waters 510 HPLC-pump (Eschborn, Germany) and a Gynkotek SP-6 UV-detector (Germering, Germany), operating at 280 nm and 5 mL min^–1^. Eluted liquids were collected in fractions of 10 mL with a fraction collector (Labomatic, Labocol Vario 4000, Allschwil, Switzerland). Chromatograms were recorded on a plotter (Shimadzu, C-R6A Chromatopac, Duisburg, Germany). One mL of dissolved sample was injected for a run. Separations were run with isocratic eluents of methanol/water (4/6, v/v) for the phloretin adduct and (1/9, v/v) for the trihydroxyacetophenone adduct with 0.8 μL mL^–1^ formic acid, respectively. The solvent of the collected fractions was evaporated under reduced pressure at 30 °C. The product was yielded as a white (phloretin adduct) or pale brown (trihydroxyacetophenone adduct) amorphous solid and stored at 4 °C. The yield was about 15–20% with respect to the phenolic compound.

### Aerated Apple Incubations

Edible parts of apples were shredded and mixed with water (1:1) for better stirring. Samples were incubated in a centrifuge tube, kept in a shaker at 37 °C under the exclusion of light, and worked up in the same way as the polyphenol incubations. For HPLC analysis, 0.5 mL of methanol was added to 1 mL of the sample to avoid analyte adsorption to the matrix. Samples were centrifuged prior to injection, and the supernatant was used for analysis.

### Stability of Dihydrocoumaric Acid and Phloretin Ascorbyl Adduct

0.5 mM *p*-dihydrocoumaric acid or phloretin ascorbyl adduct was dissolved in 2 mL of phosphate buffer (0.1 M, pH7) or added to 2 g of fresh or commercial apple puree and incubated in screw cap vials for 24 h at 37 °C in the absence of light. Phosphate-buffered samples were directly injected. Apple puree samples were mixed with 0.5 mL methanol to avoid adsorption, centrifuged prior to injection and the supernatant was used for analysis.

### High-Performance Liquid Chromatography–Diode Array Detection (HPLC–DAD)

For phenol incubations and apple puree analyses, a Jasco PU-2080 Plus quaternary gradient pump with a degasser (DG2080–54), a quaternary gradient mixer (LG 2080–02), a multiwavelength detector (MD-2015 Plus) (Jasco, Gross-Umstadt, Germany), a Waters 717 plus autosampler (Eschborn, Germany), and a column oven (Techlab Jet Stream np K-3, Erkerode, Germany) was used. Chromatographic separations were performed on stainless-steel columns (Vydac CRT, 201TP54, 250 × 4.6 mm, RP-18, 5 μm, Hesperia, CA, U.S.A.) by using a flow rate of 1.0 mL min^–1^. The column temperature was always 22 °C. The mobile phase consisted of water (solvent A) and MeOH (solvent B), and to both solvents (A and B) was added 0.8 mL L^–1^ formic acid. Samples were analyzed using a gradient system: samples were injected at 0.5% B. The gradient was kept at 0.5% B for 9 min and then changed linearly to 10% B in 1 min, to 30% B in 1 min, to 65% B in 17 min, then to 100% B in 1 min and held for 4 min. The gradient was changed linear back to 0.5% B in 2 min and held for 5 min. The effluent was monitored at 245, 280, 285, and 320 nm. Retention times were ascorbic acid *t*_R_ = 4.4 min, phloretin *t*_R_ = 27.1 min, phloridzin *t*_R_ = 21.4 min, phloretin ascorbyl adduct *t*_R_ = 22.5 min, 2,4,6-trihydroxyacetophenone *t*_R_ = 20.3 min, and 2,4,6-trihydroxyacetophenone ascorbyl adduct *t*_R_ = 16.5 min. For quantitation an external calibration based on standard solutions of authentic references dissolved in the same solvent as the sample was used.

### High-Performance Liquid Chromatography–Fluorescence Detection (HPLC–FLD)

For DMN-EP and DPA-EP analyses, a Jasco PU-980 Plus quaternary gradient pump with a degasser (DG-2080–53), ternary gradient mixer (LG 980–02), autosampler (851-AS), column oven (Jetstream 51246) and fluorescence detector (FP-4020) was used. Chromatographic separations were performed on stainless steel columns (Vydac CRT, 201TP54, 250 × 4.6 mm, RP-18, 5 μm) using a flow rate of 1.0 mL min^–1^. The column temperature was always 22 °C. The mobile phase consisted of water (solvent A) and acetonitrile (solvent B), and to both solvents (A and B) was added 0.8 mL L^–1^ formic acid. Samples were analyzed using a gradient system: For DPA and DPA-EP analysis samples were injected at 20% B. The gradient was kept at 20% B for 3 min and then changed linearly to 100% B in 30.5 min and held for 10 min. The gradient was changed linear back to 20% B in 1.5 min and held for 15 min. The effluent was monitored with 262 nm as the excitation wavelength and 422 nm as the emission wavelength. Retention times were: DPA-EP *t*_R_ = 29.5 min, DPA *t*_R_ = 33.2 min. For DMN and DMN-EP analysis samples were injected at 20% B. The gradient was kept at 20% B for 1 min and then changed linearly to 50% B in 22 min, then to 100% B in 0.5 min and held for 10 min. The gradient was changed linearly back to 20% B in 1.5 min and held for 25 min. The effluent was monitored with 230 nm as the excitation wavelength and 336 nm as the emission wavelength. DMN-EP *t*_R_ = 15.8 min, DMN *t*_R_ = 29.1 min.

### Coupled High Performance Liquid Chromatography–Mass Spectrometry (HPLC-MS)

For HPLC-MS, a Jasco PU-2080 Plus quaternary gradient pump with a degasser (DG-2080–54), quaternary gradient mixer (LG 2080–04), AS-2057 Plus autosampler set at 4 °C, and a column oven (Jasco Jetstream II) set at 22 °C was used. Chromatographic separations were performed on a stainless-steel column (Vydac CRT, 201TP54, 250 × 4.6 mm; RP-18, 5 μm).

#### Detection of Singlet Oxygen Endoperoxides

A flow rate of 0.5 mL min^–1^ was used. The mobile phase consisted of water (solvent A) and acetonitrile (solvent B), and to both solvents (A and B) 0.8 mL L^–1^ formic acid was added. Samples were analyzed using a gradient system: samples were injected at 20% B. The gradient was kept at 20% B for 5 min and then changed linearly to 100% B in 13 min and held for 22 min. The gradient was changed linearly back to 20% B in 5 min and held for 15 min. DPA-EP *t*_R_ = 24.4 min, DPA *t*_R_ = 26.3 min. Mass analyses were conducted on an API 4000 QTrap LC-MS/MS system (AB Sciex, Concord, ON, Canada) equipped with a turbo ion spray source using atmospheric pressure chemical ionization (APCI) in positive mode. The MS parameters were optimized and set as follows: an ion spray voltage of 5000 V, nebulizing gas flow of 70 mL min^–1^, heating gas of 80 mL min^–1^ at 250 °C, curtain gas of 40 mL min^–1^, declustering potential (DP) of 80 V, entrance potential (EP) of 10 V, collision energy (CE) of 28 V, and cell exit potential (CXP) of 16 V, and collision-induced dissociation (CID) was conducted for *m*/*z* 363 (M + H)^+^.

#### Detection of the Phloretin Ascorbyl Adduct

A flow rate of 1.0 mL min^–1^ was used. The eluents and gradient program were identical to those used for HPLC-DAD analysis. The mass analyses were conducted on the above LC-MS/MS system using electrospray ionization (ESI) in negative mode: a sprayer capillary voltage of −4.5 kV, nebulizing gas flow of 70 mL min^–1^, heating gas of 80 mL min^–1^ at 650 °C, curtain gas of 40 mL min^–1^, and an entrance potential of −10 V. For full scan analysis declustering potential was set at −30 V. Collision induced dissociation analysis were performed at medium adjustment. Optimized mass spectrometric parameters were as follows: *p*-dihydrocoumaric acid: *t*_R_ = 17.2 min, *m*/*z* 165.0/121.0 [DP, −75 V; CE, −15 V; CXP, −4 V], *m*/*z* 165.0/119.0 [DP, −75 V; CE, −16 V; CXP, −2 V], *m*/*z* 165.0/93.0 [DP, −75 V; CE, −23 V; CXP, −7 V], *m*/*z* 165.0/59.0 [DP, −75 V; CE, −23 V; CXP, −7 V], phloretin: *t*_R_ = 25.6 min, *m*/*z* 273.0/167.0 [DP, −42 V; CE, −24 V; CXP, −10 V], *m*/*z* 273.0/123.0 [DP, −42 V; CE, −35 V; CXP, −8 V], *m*/*z* 273.0/119.0 [DP, −42 V; CE, −35 V; CXP, −8 V], *m*/*z* 273.0/81.0 [DP, −42 V; CE, −46 V; CXP, −11 V], phloretin ascorbyl adduct: *t*_R_ = 22.8 min, *m*/*z* 419.0/299.0 [DP, −20 V; CE, −28 V; CXP, −14 V], *m*/*z* 419.0/273.0 [DP, −20 V; CE, −30 V; CXP, −15 V], *m*/*z* 419.0/209.0 [DP, −20 V; CE, −42 V; CXP, −9 V], *m*/*z* 419.0/167.0 [DP, −20 V; CE, −46 V; CXP, −7 V].

### Gas Chromatography–Flame Ionization Detector (GC-FID)

A Nexis GC-2030 gas chromatograph (Shimadzu, Duisburg, Germany) equipped with a Shimadzu autosampler (AOC-20 Plus Series) and an FID was used with helium 4.6 as a carrier gas in constant-flow mode (linear velocity of 25.2 cm/s). Samples (1 μL) were injected to a split–splitless injector at 220 °C (Split ratio of 19) and separated on a HP-5 capillary column (30 m × 0.32 mm × 0.25 μm, Agilent Technologies, Santa Clara, CA, U.S.A.). The detector was set at 300 °C. The GC oven temperature was started at 80 °C, raised to 200 °C (8 K/min) and then to 270 °C (10 K/min), and held for 10 min. The total run time was 32 min: *p-*dihydrocoumaric acid *t*_R_ = 16.6 min, dihydroisoferulic acid *t*_R_ = 18.1 min, *p*-coumaric acid *t*_R_ = 18.7 min, and dihydrocaffeic acid *t*_R_ = 18.8 min. For quantitation, an external calibration based on standard solutions of authentic references was used.

### Coupled Gas Chromatography–Mass Spectrometry (GC-MS)

A Thermo Finnigan Trace GC Ultra coupled to a Thermo Finnigan Trace DSQ instrument (Thermo Fischer Scientific GmbH, Dreieich, Germany) was used with helium 5.0 as a carrier gas in constant-flow mode (linear velocity of 35.0 cm/s). Samples (1 μL) were injected into a split–splitless injector at 220 °C (Split ratio of 19) and separated on a DB-5MS capillary column (30 m × 0.25 mm × 0.25 μm + 10 m Guard, Agilent Technologies, Santa Clara, CA, U.S.A.). MS conditions were as follows: 70 eV with electron-impact ionization (source temperature of 230 °C and emission current of 80 mA) in full-scan mode (mass range of *m*/*z* 50–650). The oven temperature program was identical to GC-FID: dihydrocoumaric acid *t*_R_ = 16.7 min, dihydroisoferulic acid *t*_R_ = 18.2 min, *p*-coumaric acid *t*_R_ = 18.8 min, dihydrocaffeic acid *t*_R_ = 18.9 min.

### High Resolution Mass Determination (HR-MS)

Negative-ion high-resolution electrospray ionization (ESI) mass spectra were obtained from a TripleToF 6600–1 mass spectrometer (Sciex, Darmstadt, Germany) equipped with a heated ESI-DuoSpray ion source and was controlled by Analyst 1.7.1 TF software (Sciex). The ESI source operation parameters were as follows: ion spray voltage of 3.7 kV; nebulizing gas: 60 psi, source temperature of 450 °C; drying gas: 70 psi, curtain gas: 35 psi. Data acquisition was performed in the MS^1^-ToF mode, scanned from 100 to 1500 Da with an accumulation time of 50 ms.

### Nuclear Magnetic Resonance Spectroscopy (NMR)

NMR spectra were recorded on a Varian VXR 400 spectrometer operating at 400 MHz for ^1^H and 100 MHz for ^13^C. SiMe_4_ was used as a reference for calibrating the chemical shift.

### Statistical Analysis

Quantitation was performed in triplicates. Limit of detection (LOD) and limit of quantitation (LOQ) were calculated at signal-to-noise ratios of 3 and 10, respectively. LOD/LOQs were calculated as mol % fragmentation yields from 0.5 mmol dihydrochalcone: dihydrocaffeic acid: 1.2 × 10^–4^%/4.0 × 10^–4^%; *p*-dihydrocoumaric acid: 2.3 × 10^–4^%/7.6 × 10^–4^%; and dihydroisoferulic acid: 3.2 × 10^–4^%/10.6 × 10^–4^%.

## Results and Discussion

### Oxidative Degradation of Dihydrochalcones

**Figure 1:**
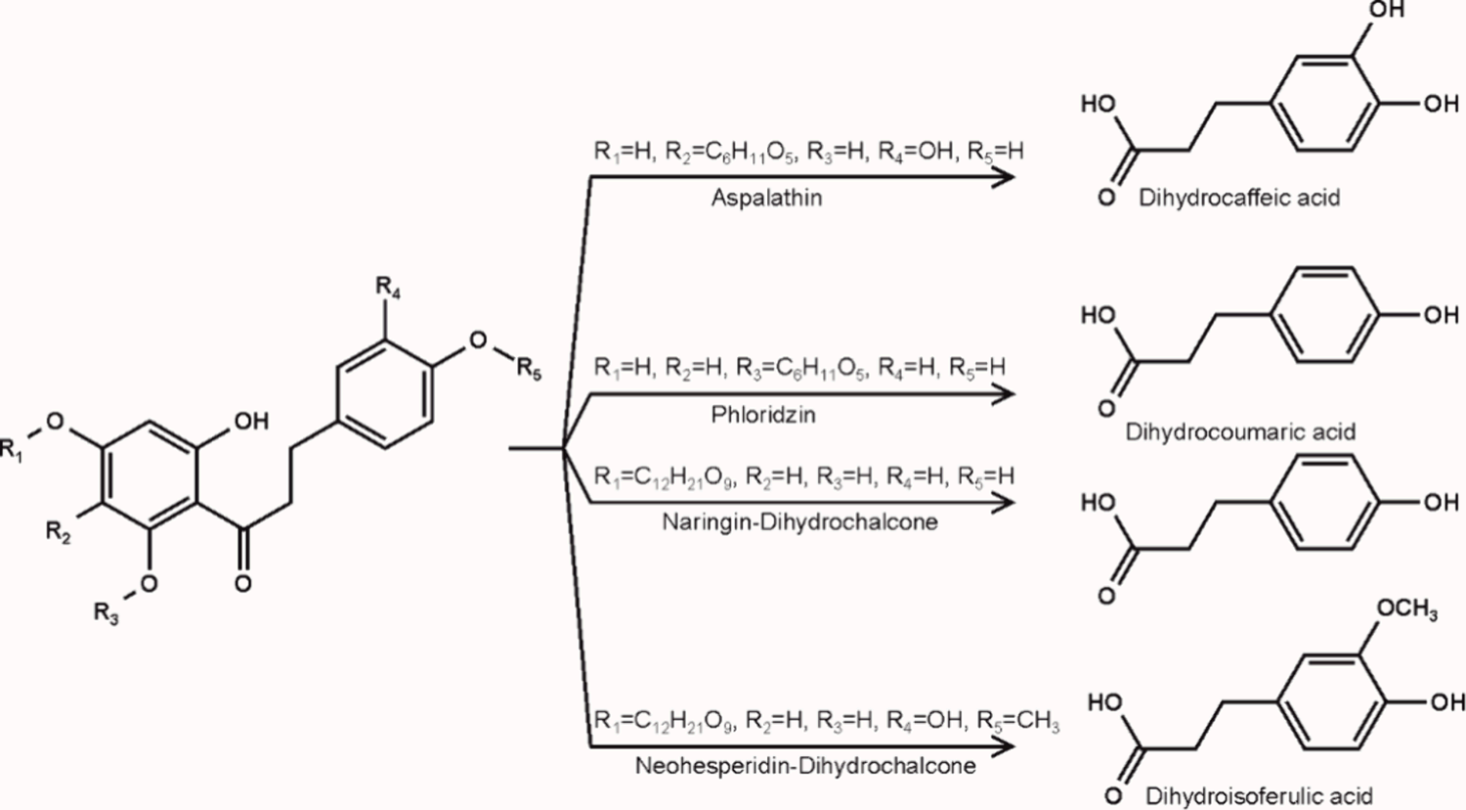
Formation of dihydrocinnamic acids from dihydrochalcones triggered by singlet oxygen via oxidative rearrangement-fragmentation.

**Figure 2:**
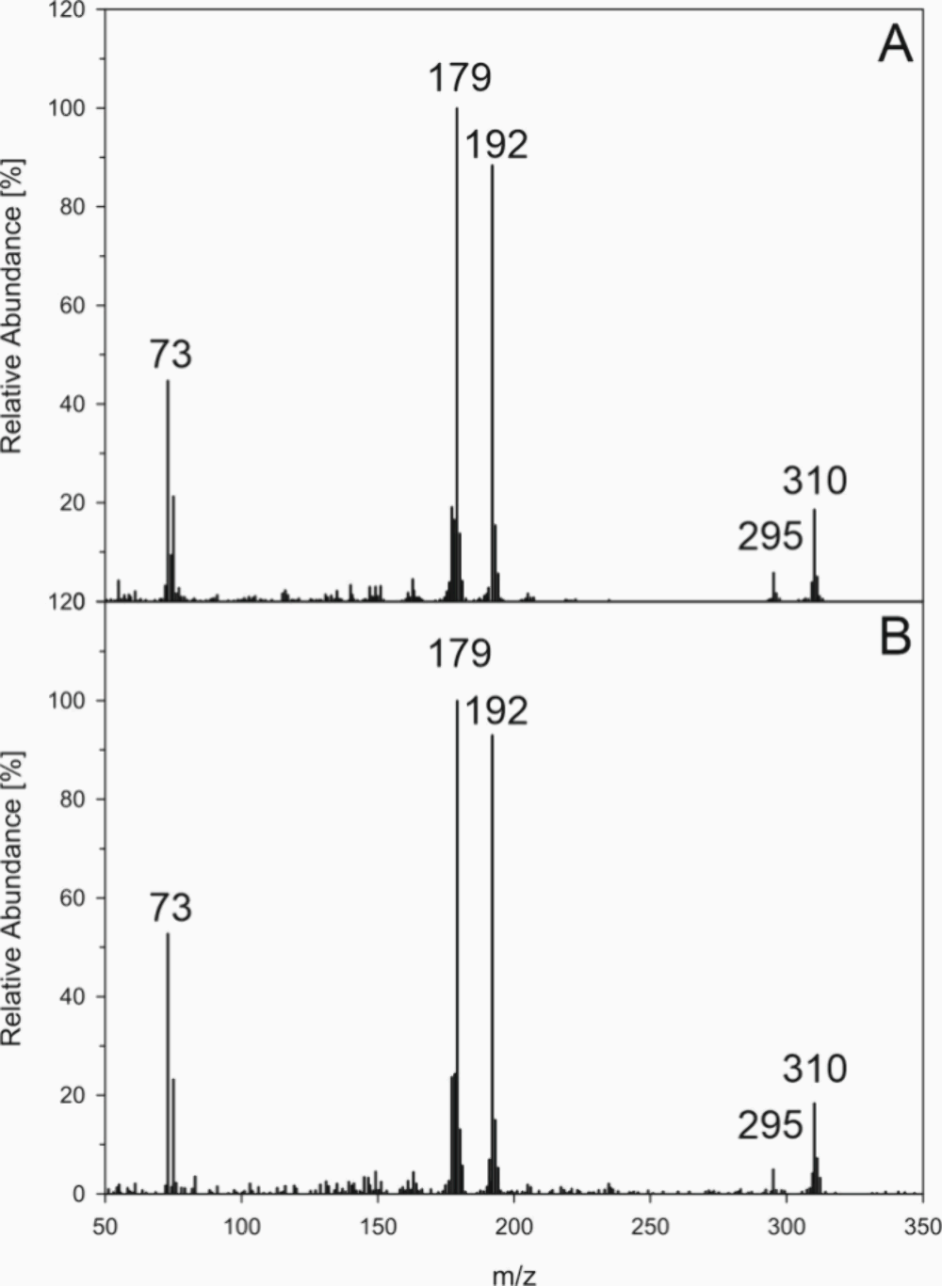
Incubation of 0.5 mM phloridzin with 2 mM DMN-EP at 37 °C and pH 7 under aeration. Verification of *p-*dihydrocoumaric acid as the trimethylsilyl ether by GC-MS, (A) authentic reference standard; (B) incubation workup.

To gain deeper insight into the singlet oxygen-triggered oxidative fragmentation of dihydrochalcones, the degradation of aspalathin and phloridzin was reinvestigated and compared to the sweeteners naringin-dihydrochalcone and neohesperidin-dihydrochalcone, which are derived from the flavanones naringin and hesperidin (Figure 1). After 24 h of incubation under aerated conditions without the addition of a singlet oxygen source, aspalathin was shown to initialize its own oxidative fragmentation to the cleavage product dihydrocaffeic acid, while no cleavage of phloridzin to *p*-dihydrocoumaric acid was observed (Table 1). This was in line with our previous results reported by Mertens et al.,^12^ who postulated aspalathin to intrinsically generate singlet oxygen under aerated conditions. The structural element required was shown to be the catechol structure located at the B-ring. Phloridzin which exhibits a monohydroxy moiety at the B-ring was not able to generate singlet oxygen, and thus, no oxidative fragmentation was monitored. Consequently, also naringin-dihydrochalcone and neohesperidin-dihydrochalcone gave no fragmentation, due to the lack of an *o*-hydroquinone structure. Neither *p*-dihydrocoumaric acid nor dihydroisoferulic acid were detected. Next, phloridzin, naringin-dihydrochalcone, and neohesperidin-dihydrochalcone were incubated with aspalathin as the singlet oxygen generator under aerated and deaerated conditions, respectively. As expected, aerated coincubations always yielded the respective dihydrocinnamic acid for aspalathin. However, dihydrocaffeic acid was formed in comparable yields to the now-triggered degradation products *p*-dihydrocoumaric acid and dihydroisoferulic acid up to 1.9 mol %, respectively. These cleavage products were unequivocally verified by comparison to authentic reference standards via GC-MS. Under deaeration, the formation of the cleavage products was strongly inhibited, showing the importance of molecular oxygen for the generation of singlet oxygen. The singlet oxygen-triggered degradation mechanism was further confirmed by using dimethylnaphthalene endoperoxide (DMN-EP) as the singlet oxygen source in deaerated single incubations of aspalathin or phloridzin. Both incubations resulted in the corresponding dihydrocinnamic acids. Figure 2 shows the detection of the phloridzin degradation product *p*-dihydrocoumaric acid, which was unequivocally verified after silylation via GC-MS by comparison to an authentic reference standard. Cleavage of aspalathin occurred 3-fold higher than that of phloridzin in the DMN-EP-containing incubations. This observance was explained through partial quenching of singlet oxygen by water, which results in triplet oxygen,^23^ mimicking incubations that resembled those under aerated conditions in which aspalathin generated singlet oxygen endogenously. This notion was underlined by the intense color formation in deaerated aspalathin samples with DMN-EP, equaling the orange color emerging as reported for aerated aspalathin incubations.

**Table 1:** Formation of Dihydrocinnamic Acids from Fragmentation of Different Dihydrochalcones (0.5 mM) without and with Different Singlet Oxygen Sources (24 h, 37 °C, pH 7, Exclusion of Light, mol %)

	(aerated)	addition of				
		2 mM aspalathin (aerated)	2 mM aspalathin (deaerated)	0.5 mM H_2_O_2_ (aerated)	0.5 mM H_2_O_2_ (deaerated)	2 mM ascorbic acid (aerated)
Aspalathin	0.14 ± 0.03%	1.80 ± 0.06%	0.22 ± 0.04%	1.26 ± 0.04%	1.62 ± 0.06%	n.a.^a^
Phloridzin	<LOD	1.94 ± 0.01%	<LOD	2.16 ± 0.09%	<LOQ	2.11 ± 0.09%
Naringin-Dihydrochalcone	<LOD	1.93 ± 0.09%	<LOD	1.55 ± 0.03%	<LOQ	1.06 ± 0.05%
Neohesperidin-Dihydrochalcone	<LOD	1.65 ± 0.02%	<LOD	1.54 ± 0.01%	<LOQ	1.29 ± 0.11%

a Not analyzed.

To further evaluate the singlet oxygen-triggered mechanism, hydrogen peroxide and ascorbic acid were chosen as singlet oxygen generators. Ascorbic acid has been demonstrated to generate singlet oxygen^24^ and is abundant in many foods, due to natural occurrence or addition to extend shelf life. Hydrogen peroxide is a common metabolite in many biotic and abiotic pathways and was also expected to occur in aspalathin samples, for it is known to be generated by flavonoids containing either a catechol or a pyrogallol structure.^25^ Hydrogen peroxide has been shown to function as a precursor for singlet oxygen generation in the presence of redox-active metal ions such as copper^24^ or molybdate ions.^26^ Indeed, in our previous studies, we used molybdate ions to catalyze the generation of singlet oxygen from hydrogen peroxide.^12^ However, even in the absence of molybdate ions, the oxidative fragmentation took place (Table 1). Singlet oxygen can be generated from reaction of hydrogen peroxide with superoxide anion or hypochlorite.^27^ A spontaneous disproportionation of hydrogen peroxide to give singlet oxygen has likewise been reported,^28^ however the amounts formed are small.^29^ Oxidative fragmentation of dihydrochalcones through hydrogen peroxide as the reactive species was ruled out based on the deaerated hydrogen peroxide incubations. No formation of the corresponding dihydrocinnamic acids occurred for phloridzin, naringin-dihydrochalcone, and neohesperidin-dihydrochalcone, proving hydrogen peroxide alone is unable to induce the oxidative rearrangement-fragmentation and confirming the necessity of a catalytic activation to give singlet oxygen. In contrast, aspalathin was shown to give dihydrocaffeic acid even in deaeration when hydrogen peroxide was present. The detection of the fragmentation product also means that small amounts of aspalathin can initiate the formation of singlet oxygen from hydrogen peroxide even in the absence of molecular oxygen. This phenomenon must be explained by the proven extremely high redox activity of aspalathin due to the combination of a B-ring catechol element next to the dihydro moiety enabling the required single electron redox transfers.^30,31^ The use of the strong chelator diethylenetriaminepentaacetic acid under deaerated conditions could lead to the notion that under aeration, traces of transition metals might catalyze singlet oxygen formation from hydrogen peroxide. This would also entail the generation of hydroxyl radicals in a Fenton reaction.^31^ However, as the anticipated hydroxylated products were not found, this reaction can be outruled. Obviously, unknown alternative pathways must exist to lead to singlet oxygen from hydrogen peroxide from phenolic compounds comprising B-ring monohydroxy or o-hydroxymethoxy moieties under aeration. Overall, aerated incubations with 2 mM ascorbic acid or aspalathin gave comparable fragmentation rates to the samples in which 0.5 mM hydrogen peroxide as the singlet oxygen precursor was added.

### Singlet Oxygen Detection in Aerated Incubations

**Figure 3:**
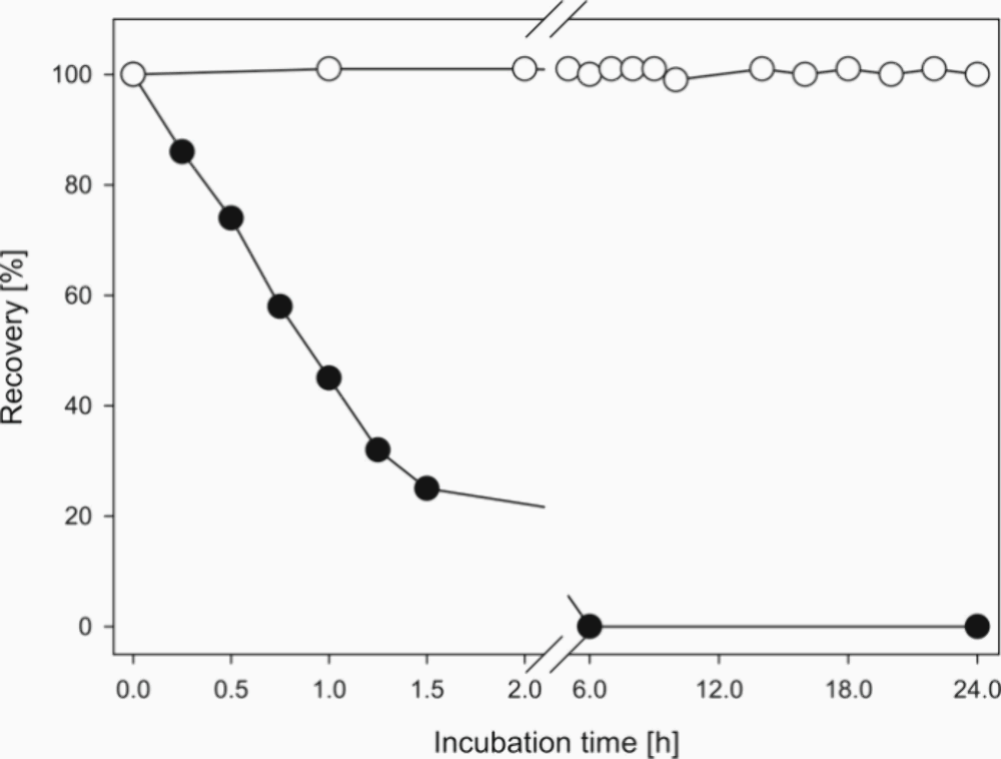
Stability of DMN-EP (●) and DPA-EP (○) at 37 °C and pH 7 under aeration.

**Figure 4:**
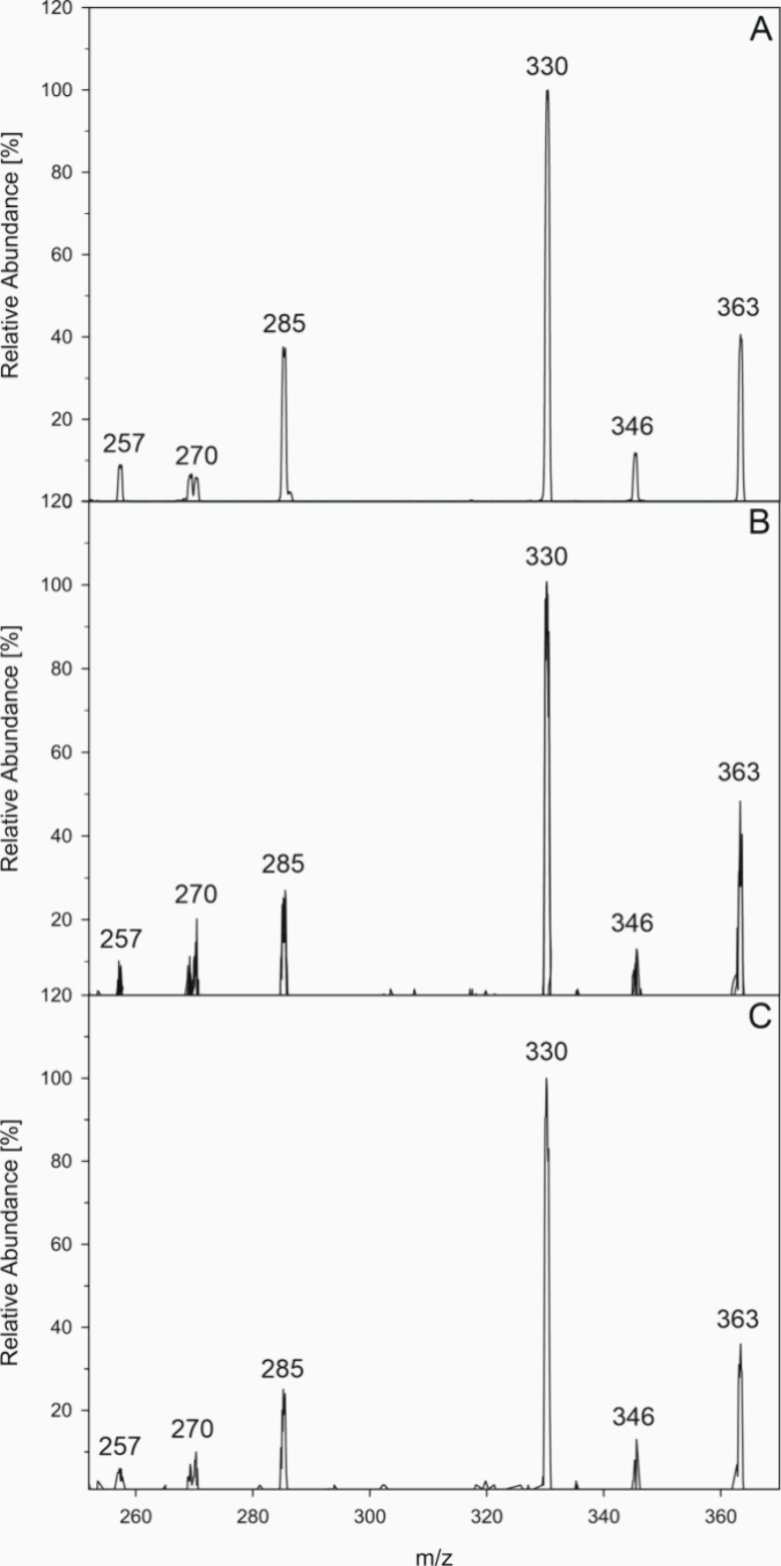
Formation of specific singlet oxygen endoperoxids in aspalathin and ascorbic acid incubations (37 °C, pH 7, aeration). Verification of DPA-EP by collision induced dissociation (CID) of *m*/*z* 363 (M + H)^+^ via HPLC(+)-APCI-MS^2^, (A) authentic reference standard; (B) aspalathin incubation; (C) ascorbic acid incubation.

The ability of flavonoids to generate singlet oxygen has been investigated in numerous studies. However, most reports are based on photochemical singlet oxygen formation.^32,33^ Few data have been published on singlet oxygen formation by flavonoids under the exclusion of light, though some of these compounds are expected to produce singlet oxygen endogenously due to their published ability to produce both hydrogen peroxide^25^ and superoxide anion.^31^ In our previous studies, we reported the singlet oxygen formation of aspalathin. The detection of singlet oxygen was argued by the analysis of specific oxidized products.^12^ Although singlet oxygen emits chemiluminescence at 1275 nm,^13^ the more common detection method is the use of a chemical trap, which reacts specifically with singlet oxygen. Typical structures are anthracene or naphthalene derivatives,^14^ which give endoperoxides specific for the singlet oxygen reaction. On the contrary, these endoperoxides can also be used to specifically release singlet oxygen. Turro and Chow^14^ reported thermolysis of naphthalene endoperoxides at 35 °C, while higher temperatures were needed to cleave anthracene endoperoxides. Importantly, all measurements were conducted in organic solvents. With the present report, we confirmed the higher stability of 9,10-diphenylanthracene-9,10-endoperoxide (DPA-EP) compared to DMN-EP by incubating both endoperoxides under the aqueous conditions of the above incubation setup. As seen in Figure 3 DMN-EP entirely released singlet oxygen within 6 h. It can therefore be used as a specific provider to trigger singlet oxygen-mediated reactions, as we see in Figure 2. In contrast, DPA-EP did not undergo degradation at 37 °C but remained fully stable under the given mild conditions. Thus, DPA can be used as a chemical trap for singlet oxygen, with the generated DPA-EP accumulating during the incubation. Consequently, DPA was incubated with aspalathin or ascorbic acid as a singlet oxygen generator, and DPA-EP was confirmed using HPLC/APCI(+)-MS^2^ experiments. To rule out spontaneous conversion of DPA to DPA-EP by other reactions, blank samples without phenols were conducted in the same way. As shown in Figure 4 MS-CID experiments both for aspalathin (4B) and for ascorbic acid incubations (4C) gave mass spectra virtually identical to those of an authentic DPA-EP standard. Taken together we verified for the first time singlet oxygen generation in aerated aspalathin and ascorbic acid samples under mild aqueous conditions and exclusion of light.

### Oxidative Degradation of Flavanones

To further investigate singlet oxygen-triggered oxidative fragmentations, also the flavanones naringenin, hesperetin, and eriodictyol were incubated under the same conditions as the dihydrochalcones with either aspalathin or hydrogen peroxide as the singlet oxygen source. Flavanones can reversibly isomerize into the corresponding chalcones,^34^which, in parallel to the above dihydrochalcone reactions, should fragment in the presence of singlet oxygen. However, neither *p*-coumaric acid nor caffeic acid or isoferulic acid were detected after 24 h of incubation at pH 7. This observation must be attributed to the known isomerization equilibrium between flavanone and chalcone, which is almost exclusively on the side of the flavanone between pH 0 and 10,^34^ which is chemically inert to singlet oxygen.^35^ This conclusion was confirmed by reacting naringenin at pH 12 with ascorbic acid or hydrogen peroxide to induce ring opening of the chalcone. As expected, *p-*coumaric acid was now detected. However, flavanones were not further investigated, as the required strong alkaline conditions have no relevance in food matrices. When the natural chalcone xanthohumol was incubated, *p-*coumaric acid was indeed confirmed as an oxidative cleavage product at pH 7. This was expected, as the chalcone-flavanone (isoxanthohumol) equilibrium due to the isoprenic alkylated A-ring substitution is hindered and provides substantial chalcone amounts at food-relevant pH values. Thus, the above singlet oxygen-mediated fragmentation reactions for dihydrochalcones can also be applied to chalcones.

### Ascorbic Acid Phloretin Incubations

**Figure 5:**
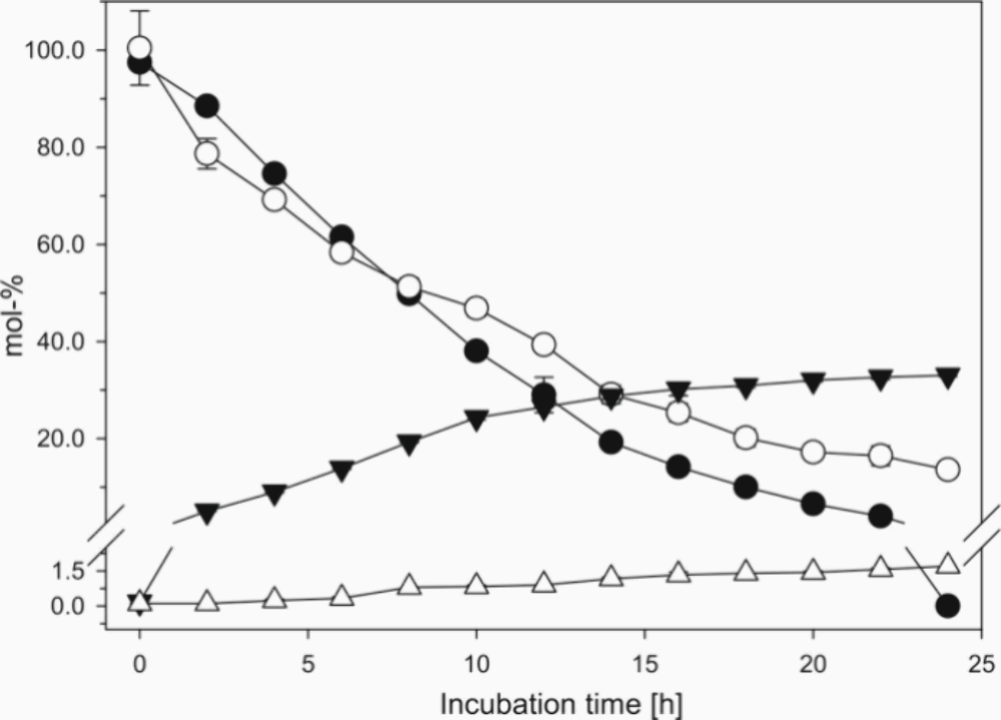
Incubation of 0.5 mM phloretin in the presence of 2.0 mM ascorbic acid (37 °C, pH 7, aeration): phloretin (○), ascorbic acid (●), *p*-dihydrocoumaric acid (**Δ**), and phloretin ascorbyl adduct (**▼**).

As ascorbic acid is both a singlet oxygen generator and abundant in food matrices, ascorbic acid was chosen for further mechanistic investigations on the oxidative degradation of phloretin, whose glycosides constitute the major polyphenolic content of apples. Model incubations showed an almost similar degradation of phloretin and ascorbic acid of about 90 mol % at 20 h paralleled by the expected emergence of *p*-dihydrocoumaric acid (1.5 mol %) as in above phloridzin reactions (Figure 5). Simultaneously, a novel ascorbyl adduct was detectable in significant amounts, reaching concentrations of 31 mol % after 24 h. Incubations starting with phloridzin or *p*-dihydrocoumaric acid gave no adduct formation, strongly pointing toward the involvement of the A-ring in the reaction.

### Isolation and Elucidation of the Phloretin Ascorbyl Adduct

Because of the high formation rate, it was possible to isolate the novel ascorbyl adduct from model incubations by ethyl acetate extraction followed by reversed-phase chromatography as a white powder. It showed an absorption maximum at 266 nm and high-resolution mass spectrometry with negative ionization gave a peak at *m*/*z* 419.0995 [M – H]^−^, which corresponds to the elemental composition of C_20_H_20_O_10_ with 2.7 ppm accuracy. This indicated a phloretin ascorbyl adduct with the loss of one carbon atom. The structure of the isolated compound was unequivocally verified using ^1^H NMR and ^13^C NMR measurements, as well as heteronuclear single quantum coherence (HSQC), heteronuclear multiple bond correlation (HMBC), and homonuclear correlation spectroscopy (H,H COSY) techniques (Table 2). ^1^H as well as ^13^C measurements yielded more signals than expected, with sets of two signals having nearly identical shifts, respectively. This was attributed to the existence of two diastereoisomers with differing stereogenic centers located in the newly formed five-membered ring (C-12, isomeric ratio based on ^1^H NMR). The signals of the B-ring and the bridging C_3_-moiety compared to the precursor phloretin were virtually unchanged in, both, ^1^H and ^13^C spectra, showing no alteration had occurred at these positions, again pointing toward the participation of the A-ring in the reaction. This was in line with the above-observed absence of adduct formation in phloridzin and *p*-dihydrocoumaric acid incubations. The NMR data further substantiated the loss of one aromatic proton in the A-ring, while no new proton signals were observed. The remaining proton signals were allocated to the ascorbic acid side chain, showing the adduct formation is taking place at the former reductone moiety. Instead, two new signals of C-11 (194.9 ppm) and C-12 (107.0 ppm/108.4 ppm) were observed, which showed an HMBC correlation to the aromatic proton H-8 (5.90 ppm), thus indicating the interaction to the A-ring. The carbon resonance at 194.9 ppm was identified as a ketone, while the carbon at 107.0 ppm/108.4 ppm was classified by the typical resonance of a hemiacetal and attributed to the location of the differing stereogenic center, also due to the comparatively high variance in the chemical shift (1.4 ppm) between the two isomeric signals. In theory, ring closure could also occur via the hydroxy groups in *o*-position to the chalcone acyl substitution and result in a second set of isomers, which was not found. Obviously, this would create a major steric hindrance. The assigned closure via the *p*-substituted hydroxyl group can also be seen in slight downfield shifts of aromatic carbons C-6 and C-7, while C-9 and C-10 were almost not changed compared to a phloretin reference. Thus, the structure was unequivocally identified as 2,4,6-trihydroxy-5-[3-(4-hydroxyphenyl)propanoyl]-2-[(1*R*, 2*S*)-1,2,3-trihydroxypropyl]-1-benzofuran-3(2*H*)-one.

**Table 2:**
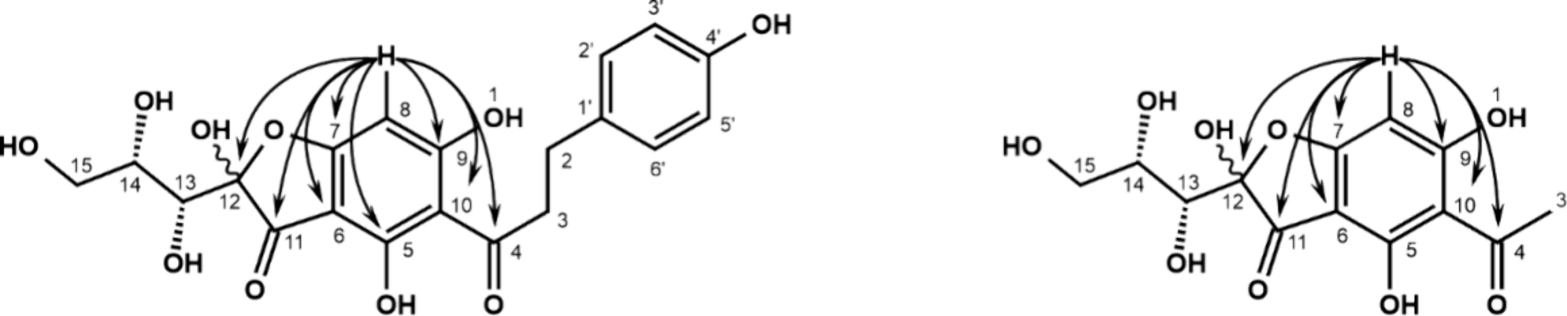
High-resolution Mass and ^1^H- and ^13^C-NMR Spectroscopic Data of the Ascorbyl Adducts of Phloretin and 2,4,6-Trihydroxyacetophenone (in CD_3_OD), Selected HMBC Correlations Are Highlighted by Arrows

HR-MS [M – H]^−^ (*m*/*z*)			419.0995		HR-MS [M – H]^−^ (*m*/*z*)			313.0572	
calc. C_20_H_19_O_10_^–^ (*m*/*z*)			419.0984		calc. C_13_H_14_O_9_^–^ (*m*/*z*)			313.0565	
C/H	isomer 1 (∼60%)		isomer 2 (∼40%)		C/H	isomer 1 (∼60%)		isomer 2 (∼40%)	
	δ^1^H [ppm]	δ ^13^C [ppm]	δ^1^H [ppm]	δ ^13^C [ppm]		δ^1^H [ppm]	δ ^13^C [ppm]	δ^1^H [ppm]	δ ^13^C [ppm]
2	3.35–3.51 (m, 2H)	44.5	3.35–3.51 (m, 2H)	45.0	2				
3	2.84–2.99 (m, 2H)	28.9	2.84–2.99 (m, 2H)	29.1	3	2.70 (s, 1H)	30.4	2.67 (s, 1H)	30.2
4		203.7		203.5	4		202.0		201.7
5		175.1		175.1	5		175.5		175.3
6		102.3		102.6	6		102.3		102.5
7		173.5		174.0	7		173.3		173.8
8	5.90 (s, 1H)	96.3	5.90 (s, 1H)	96.5	8	5.87 (s, 1H)	96.1	5.88 (s, 1H)	96.3
9		162.8		163.2	9		162.9		163.2
10		100.9		100.8	10		101.2		101.1
11		194.9		194.9	11		195.0		194.9
12		107.0		108.4	12		107.0		108.3
13	3.97 (d, 1H)	71.2	3.98 (d, 1H)	71.7	13	3.95 (d, 1H)	71.0	3.97 (d, 1H)	71.6
	^3^*J* = 3.2 Hz		^3^*J* = 2.4 Hz			^3^*J* = 2.5 Hz		^3^*J* = 2.2 Hz	
14	4.08 (dt, 1H)	69.7	4.17 (dt, 1H)	70.4	14	4.12 (m, 1H)	69.5	4.13 (m, 1H)	70.5
	^3^*J* = 3.2 Hz		^3^*J* = 2.4 Hz			^3^*J* = 2.5 Hz		^3^*J* = 2.2 Hz	
	^3^*J* = 5.8 Hz		^3^*J* = 6.3 Hz			^3^*J* = 6.5 Hz		^3^*J* = 5.7 Hz	
15	(A) 3.67 (dd, 1H)	63.3	(A/B) 3.63 (m, 2H)	62.6	15	(A) 3.66 (dd, 1H)	63.3	(A/B) 3.61 (m, 2H)	62.6
	^2^*J* = 11.0 Hz		^2^*J* = 11.0 Hz			^2^*J* = 11.0 Hz			
	^3^*J* = 5.8 Hz		^3^*J* = 6.3 Hz			^3^*J* = 6.0 Hz			
	(B) 3.63 (dd, 1H)					(B) 3.61 (dd, 1H)			
	^2^*J* = 11.0 Hz					^2^*J* = 11.0 Hz			
	^3^*J* = 5.8 Hz					^3^*J* = 6.5 Hz			
1‘		132.0		132.0	1‘				
2‘/6‘	7.10 (d, 2H)	129.1	7.09 (d, 2H)	129.0	2‘/6‘				
	^3^*J* = 8.5 Hz		^3^*J* = 8.4 Hz						
3‘/5‘	6.69 (d, 2H)	114.7	6.70 (d, 2H)	114.8	3‘/5‘				
	^3^*J* = 8.5 Hz		^3^*J* = 8.4 Hz						
4‘		155.1		155.2	4‘				

### Mechanism of the Phloretin Ascorbyl Adduct Formation

**Figure 6:**
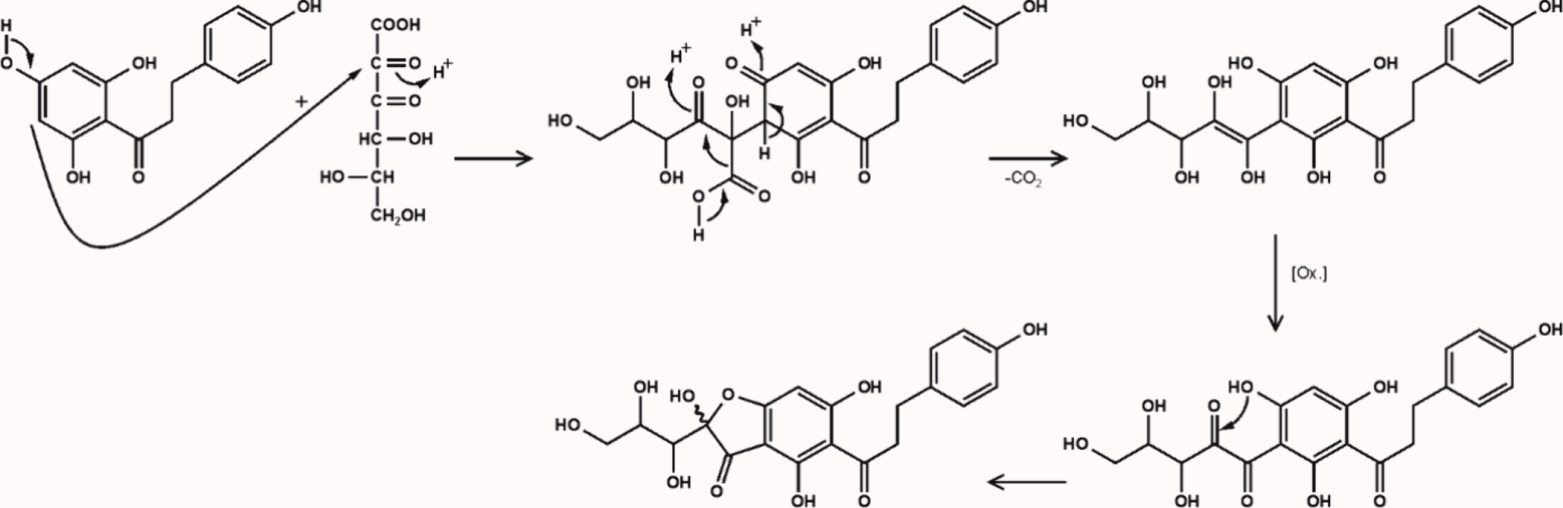
Proposed mechanism for the formation of the novel phloretin ascorbyl adduct.

On the basis of the present investigations and the knowledge so far published for the generation of adducts between phenolic compounds and reactive α-dicarbonyl structures, the following mechanism for the formation of the phloretin ascorbyl adduct was proposed (Figure 6). The initiating step is oxidation and hydrolysis of ascorbic acid to yield 2,3-diketogulonic acid^17^ which reacts by nucleophilic addition of the A-ring to the electron-deficient C-2 carbonyl position. The intermediate decarboxylates via a β-decarboxylation to result in an endiol structure that obviously due to conjugation to the rearomatized electron-rich A-ring is readily oxidized to give an α-dicarbonyl moiety. Ring closure then leads to a five-membered hemiacetal ring, thereby explaining the formation of two diastereoisomers in line with the above NMR discussion. The isolated phloretin ascorbyl adduct is a novel structure that has not yet been described, though trapping reactions of reactive α-dicarbonyl species such as glyoxal and methylglyoxal by flavonoids under physiological conditions have been reported in the literature with the A-ring shown to be the major nucleophilic site.^15,36^ The formation of flavonoid ascorbyl adducts has also been described. Hung et al.^18^ reported the entire dehydroascorbic acid molecule to be incorporated into the adduct. However, the molecular mass given differed from that isolated herein. Here, the central reaction is the β-decarboxylation of the C-1 carboxylic acid moiety. To unambiguously confirm this mechanism, incubations of phloretin and ascorbic acid were carried out using ^13^C-1 or ^13^C-2 labeled ascorbic acid. Indeed, incubations of phloretin with ^13^C-1 labeled ascorbic acid gave the ascorbyl adduct with a molar mass of *m*/*z* of 419 [M – H]^−^ and a fragmentation spectrum identical to the one from unlabeled experiments, which verified the elimination of the C-1 carbon. Incubations of phloretin with ^13^C-2 labeled ascorbic acid yielded an *m*/*z* of 420 [M – H]^−^, confirming the incorporation of the labeled carbon into the final structure, in line with the proposed formation pathway.

When comparing ascorbic acid incubations of phloretin to its *O*-glycosidic pendant phloridzin, no ascorbyl adduct was detectable in phloridzin samples. To evaluate further structural elements required for adduct generation, ascorbic acid was incubated with the flavanon naringenin and the flavonol kaempferol, respectively. However, both incubations gave no mass spectrometric signals that agreed with a similar adduct formation. Obviously, ascorbyl adduct formation seems to depend on a free phloroglucinol A-ring constitution. Therefore, naringenin was reinvestigated with ascorbic acid at pH 12, to convert the flavanone into the corresponding chalcone. Naringenin chalcone is almost identical to phloretin except for the conjugated double bond in the bridging C_3_-moiety. No adduct formation was monitored, while the oxidative fragmentation described above to *p*-coumaric acid was indeed observed. However, the lack of adduct formation might also be attributed to the rapid decomposition of ascorbic acid at such high pH values.^37^ To further evaluate the required structural elements, incubations with selected phenols with varying substitution patterns were conducted (Table 3). No adduct formation was noted in any sample containing *m*-dihydroxyphenols (2,4-dihydroxyacetophenone, 2,4-dihydroxybenzoic acid). The resorcinol structure creates a central nucleophilic carbon,^38^ whose nucleophilicity must be increased by a third electron-donating hydroxyl group. Obviously, the phenolic *m*-dihydroxy constitution alone generates too little nucleophilicity for adduct formation. However, even most 1,3,5-trihydroxy phenols showed only traces of ascorbyl adduct formation monitored by mass spectrometry (2,4,6-trihydroxybenzoic acid, 2,4,6-trihydroxytoluene, phloroglucinol). The only exception turned out to be 2,4,6-trihydroxy acetophenone with yields of more than 40%, which resembles the A-ring and bridging C_3_-moiety of phloretin. It was therefore concluded that besides the electron-donating effect caused by the three hydroxyl groups in the *o*- and *p*-position, the electron-directing effect of the acetyl moiety to the *m*-position likewise contributes to the specific nucleophilicity leading to ascorbyl adduct formation. The 2,4,6-trihydroxyacetophenone ascorbyl adduct was isolated and unequivocally verified via HR-MS and NMR (Table 2). NMR shifts were shown to be identical to those of the phloretin ascorbyl adduct, and the same diastereomeric pattern was monitored. This conclusively underlined the formation mechanism depicted in Figure 6.

**Table 3:** Formation of Adducts in Incubations of Different Phenolic Precursor Molecules (0.5 mM) with Ascorbic Acid (2 mM) after 24 h (pH 7, Aerated, Exclusion of Light)

precursor	adduct formation [mol %]
phloretin	30.8 ± 0.3
phloridzin	n.d.^a^
2,4,6-trihydroxyacetophenone	41.2 ± 0.6
2,4,6-trihydroxybenzoic acid	traces
2,4,6-trihydroxytoluene	traces
phloroglucinol	traces
2,4-dihydroxyacetophenone	n.d.^a^
2,4-dihydroxybenzoic acid	n.d.^a^

a Not detected.

### Phloretin Ascorbyl Adduct Formation in Apple Incubations

**Figure 7:**
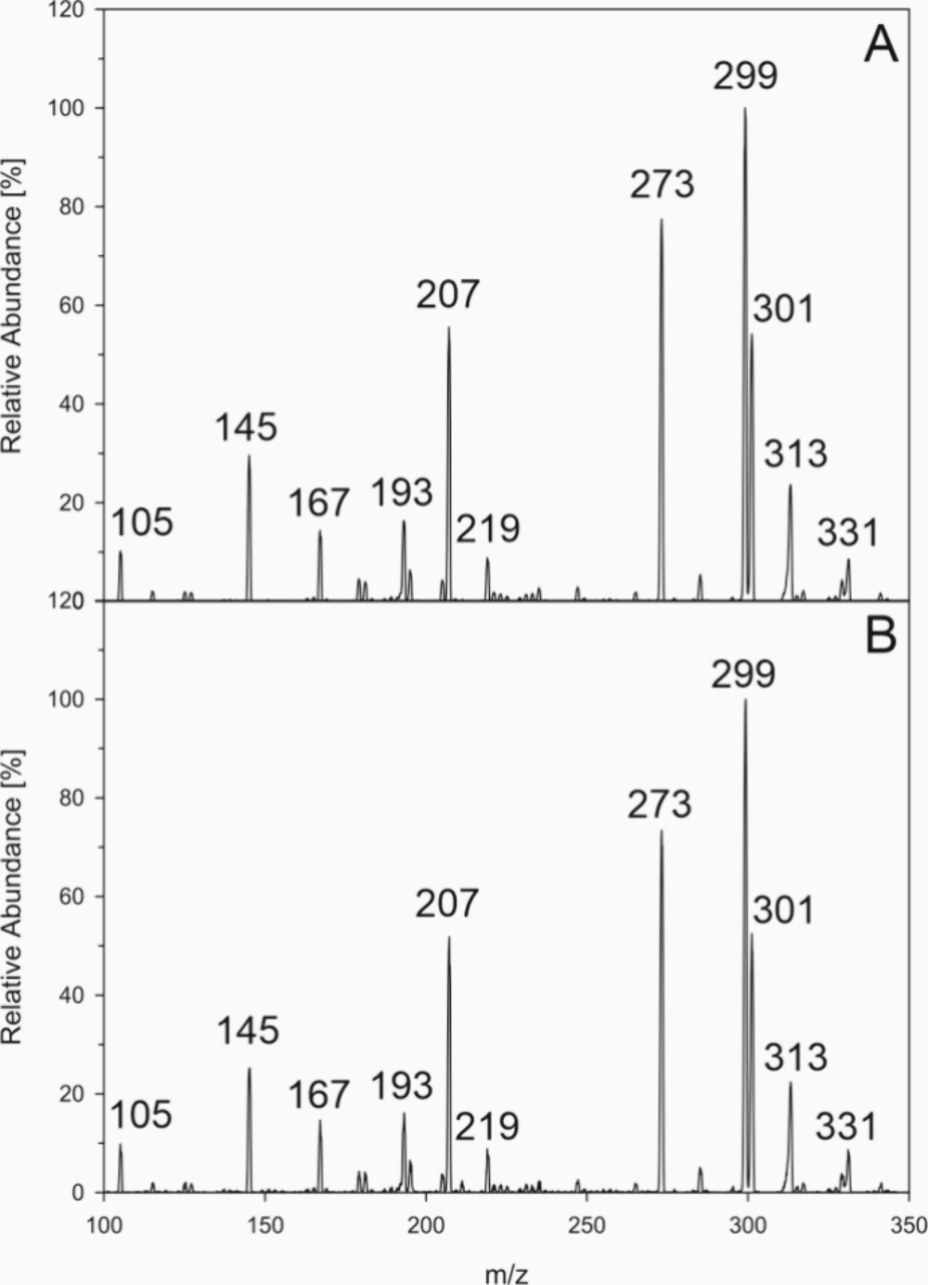
Incubation of commercial pasteurized apple puree spiked with 0.5 mM phloretin (24 h, 37 °C, aeration). Verification of the phloretin ascorbyl adduct by collision induced dissociation (CID) of *m*/*z* 419 (M – H)^−^ via LC(−)-ESI-MS^2^, (A) authentic reference standard; (B) apple puree workup.

The relevance of the phloretin ascorbyl adduct formation was tested in commercial apple samples. Apples contain phloretin in the form of *O*-glycosylated phloridzin which was shown above to be unsusceptible for adduct formation. However, when apples are eaten or chopped up, phloretin is released by hydrolases. Ascorbic acid is naturally found in apples and is prevalently also added to apple purees to prevent browning as an antioxidant. However, it has been shown that dehydroascorbic acid can indeed react with nucleophiles such as amino acids to lead to advanced glycation protein modifications.^38,39^ Here, first the stabilities of *p*-dihydrocoumaric acid and the phloretin ascorbyl adduct were tested in model incubations as well as in fresh and commercial pasteurized apple puree. Both products were completely recovered in model incubations and by around 95% in pasteurized apple purees. However, phloretin is not likely to be released from glycosides in pasteurized apple purees due to the thermal inactivation of hydrolases. In fresh apple puree samples, recovery ranged from 58% for *p*-dihydrocoumaric acid to 8% for the ascorbyl adduct within 24 h of incubation. Likewise, phloretin and ascorbic acid showed a major reduction to trace levels within only 4 h of incubation. Thus, due to the high lability of phloretin, ascorbic acid as well as the phloretin ascorbyl adduct, apple purees were spiked with phloretin and ascorbic acid to start from defined concentrations. Indeed, this led to the formation of the ascorbyl adduct unambiguously verified via LC-MS/MS experiments. The resulting mass spectrum gave virtually the same fragmentation pattern as the authentic reference. In addition, in commercial pasteurized apple purees spiked with phloretin alone, ascorbyl adduct formation occurred (Figure 7), proving that the formation of the phloretin ascorbyl adduct is taking place when both precursor molecules are present.

In summary, we succeeded for the first time in proving the intrinsic singlet oxygen generation by the dihydrochalcone asphalathin and ascorbic acid at a neutral pH in the absence of light. The singlet oxygen-induced oxidative fragmentation of dihydrochalcones was further underlined. The reaction was also transferred to flavanones when the C-ring opened to give the corresponding chalcones. In addition, a novel phloretin ascorbyl adduct was identified and proven to be generated in commercial apple samples when the precursor molecules phloretin and dehydroascorbic acid are both present. Ongoing research will now explore the impact of singlet oxygen on degradation reactions with other flavonoids.

## Abbreviations Used

AGE: advanced glycation end products
APCI: atmospheric pressure chemical ionization
CE: collision energy
CID: collision induced dissociation
CXP: cell exit potential
DMN: dimethylnaphthalene
DMN-EP: dimethylnaphthalene-endoperoxide
DP: declustering potential
DPA: diphenylanthracene
DPA-EP: diphenylanthracene-endoperoxide
ESI: electrospray ionization
EP: entrance potential
